# ExTraCT – Explainable trajectory corrections for language-based human-robot interaction using textual feature descriptions

**DOI:** 10.3389/frobt.2024.1345693

**Published:** 2024-09-23

**Authors:** J-Anne Yow, Neha Priyadarshini Garg, Manoj Ramanathan, Wei Tech Ang

**Affiliations:** ^1^ Rehabilitation Research Institute of Singapore (RRIS), Joint Research Institute by Nanyang Technological University (NTU), Agency for Science, Technology and Research (A∗STAR) and National Healthcare Group (NHG), Singapore, Singapore; ^2^ Singapore-ETH Centre, Future Health Technologies Programme, CREATE campus, Singapore, Singapore

**Keywords:** human-robot interaction, language in robotics, natural language processing, assistive robots, foundational models, large language models

## Abstract

**Introduction:**

In human-robot interaction (HRI), understanding human intent is crucial for robots to perform tasks that align with user preferences. Traditional methods that aim to modify robot trajectories based on language corrections often require extensive training to generalize across diverse objects, initial trajectories, and scenarios. This work presents ExTraCT, a modular framework designed to modify robot trajectories (and behaviour) using natural language input.

**Methods:**

Unlike traditional end-to-end learning approaches, ExTraCT separates language understanding from trajectory modification, allowing robots to adapt language corrections to new tasks–including those with complex motions like scooping–as well as various initial trajectories and object configurations without additional end-to-end training. ExTraCT leverages Large Language Models (LLMs) to semantically match language corrections to predefined trajectory modification functions, allowing the robot to make necessary adjustments to its path. This modular approach overcomes the limitations of pre-trained datasets and offers versatility across various applications.

**Results:**

Comprehensive user studies conducted in simulation and with a physical robot arm demonstrated that ExTraCT’s trajectory corrections are more accurate and preferred by users in 80% of cases compared to the baseline.

**Discussion:**

ExTraCT offers a more explainable approach to understanding language corrections, which could facilitate learning human preferences. We also demonstrated the adaptability and effectiveness of ExTraCT in a complex scenarios like assistive feeding, presenting it as a versatile solution across various HRI applications.

## 1 Introduction

**FIGURE 1:**
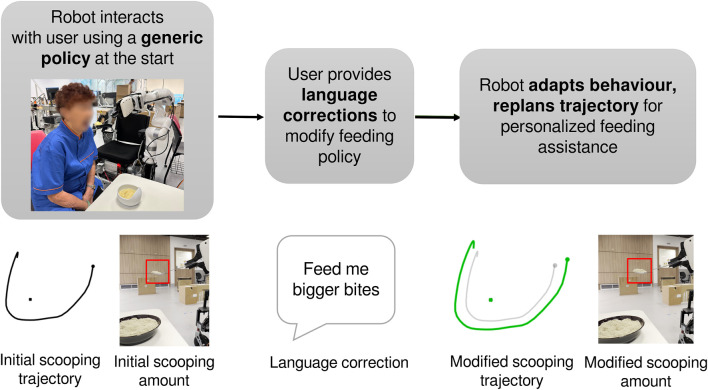
Human-robot interaction during assistive feeding. When interacting with the user for the first time, the robot executes a generic policy. Given user feedback in the form of language, the robot then modifies its behaviour to personalize its assistance.

Motion planning algorithms optimize trajectories based on predefined cost functions, which usually consider robot dynamics and environmental constraints. However, robots need to account for human preferences to assist effectively when working with humans. Human preferences can vary based on the environment and human factors; e.g., during mealtimes, users may prefer the robot to feed bigger bites when hungry but may prefer the robot to feed smaller bites when full (Figure 1); when throwing trash, some may prefer the robot to place the trash away from food items to maintain hygiene, while others may prefer it to take the shortest route for efficiency. Thus, incorporating all possible human preferences in the cost function is challenging. To address this, we explore the problem of natural language trajectory corrections, i.e., modifying a robot’s initial trajectory based on natural language corrections provided by a human. We choose natural language as it provides an intuitive and expressive way for humans to convey their preferences.

A key challenge in natural language trajectory corrections is mapping the natural language to the robot action, which is the deformed trajectory. Existing works (Broad et al., 2017; Sharma et al., 2022; Bucker et al., 2022; 2023) try to learn a direct mapping between natural language and robot trajectories or actions using offline training paradigms. To improve generalization to different objects and phrases, some works (Sharma et al., 2022; Bucker et al., 2022; 2023) leverage on foundational models, i.e., BERT (Devlin et al., 2018) and CLIP (Radford et al., 2021). However, these models struggle to generalize to varied initial trajectories and object poses due to dataset limitations. Furthermore, with a direct mapping learnt between natural language and robot action, it is difficult to understand the root cause of failures Adadi and Berrada (2018), which we believe could stem from issues in language grounding, scene understanding, or inaccuracies in trajectory deformation function.

**FIGURE 2:**
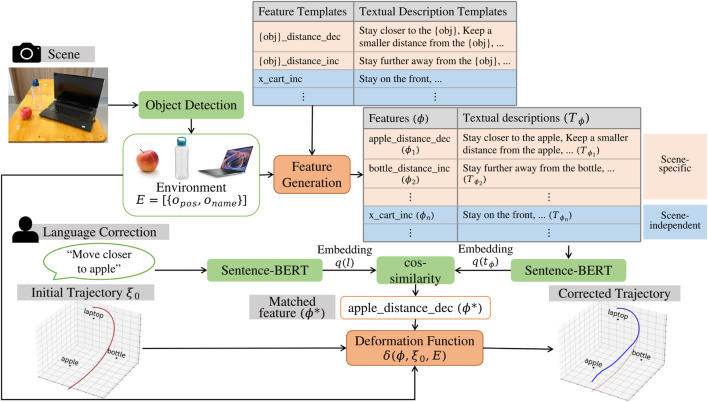
Architecture of ExTraCT. Given the objects in a scene, the features ϕ and corresponding textual descriptions $T_{\phi }$ are generated online. We obtain the embeddings of the language correction q(l) and the phrases $\left(t_{\phi }\in T_{\phi }\right)$ in the textual descriptions of the features $q\left(t_{\phi }\right)$ , and use semantic textual similarity to obtain the most similar textual description, which is mapped to feature $\phi^{*}$ . A deformation function δ is used to deform the initial trajectory $\xi_{0}$ based on the feature $\phi^{*}$ and the object positions in the environment E . A trajectory optimizer is used to ensure that the robot’s kinematic constraints are satisfied.

To address these limitations, we separate language understanding from trajectory deformation, thus enabling a more accurate interpretation of instructions. First, we match the language corrections to a short description of the change in trajectory, which we term a feature. We define a set of feature templates and their corresponding textual description templates, which are then used to generate features and their textual descriptions (Figure 2) for any given scene online. The language uttered by the user can then be mapped to the most likely feature by computing the semantic similarity between the textual descriptions of the feature and the correction uttered by the user using Large Language Models (Devlin et al., 2018; Wang et al., 2020; Brown et al., 2020; Chowdhery et al., 2022). For features that are mapped with insufficient confidence, our approach can alert the end-user instead of generating a random modified trajectory. In this work, we set the confidence threshold to be 0.6, which was determined empirically based on a small set of testing data. Note that this test data is different from the set we used in the experiments below. If the threshold is set too high, the system would alert the user too frequently that feature matching failed. Conversely, a threshold too low could lead to incorrect feature matching, i.e., matching to a feature even though the correction could be out of the feature space.

Once the most likely feature is determined, the initial trajectory can be modified based on a trajectory deformation function, allowing generalization to different object configurations and trajectories.

This separation of language understanding from trajectory deformation allows for more precise and context-aware robotic responses. By decoupling these two elements, our method can easily expand to new tasks, as shown in Section 4.5. Furthermore, it provides clearer insights into potential sources of failure, as it distinguishes between errors in language interpretation and trajectory execution. Thus, our approach improves the accuracy and generalization of language corrections and enhances the interpretability and reliability of robotic systems in executing these corrections.

While our approach is limited by the feature types in the feature templates, end-to-end training methods would encounter similar limitations when dealing with unseen features. However, they are additionally limited by the object configurations and initial trajectories used in the training data, as shown in Section 4.1. Our formulation can generalize to different object configurations and initial trajectories without training a model. Our key insight is that integrating the strengths of LLMs in handling language diversity with a hand-crafted approach for trajectory modifications bypasses the need for end-to-end training while achieving comparable or better performance. Furthermore, end-to-end training methods are data-intensive. Collecting data is challenging in the robotics domain, while generating data would require a similar amount of hand-crafted features as our proposed approach.

We evaluated our system through within-subject user studies in simulation and the real world. Our results show that our method had higher accuracy and was rated higher in approximately 80% of test cases compared to the state-of-the-art method LaTTe (Bucker et al., 2023), which also uses LLMs but was trained in an end-to-end fashion. We further analysed the failure cases of our method and showed that our method could be improved further by adding more phrases to the textual description templates.

Our contributions in this work are threefold: (1) We introduce a modular framework that integrates LLMs with trajectory deformation functions for trajectory corrections using language without end-to-end training. (2) We conducted extensive quantitative experiments on a substantial dataset, complemented by user studies comparing our method against a baseline that deforms trajectories based on language in an end-to-end fashion. (3) We demonstrate the versatility of our framework through its application to a range of tasks, including general object manipulation and assistive feeding.

## 2 Related work

There are various ways of conveying human preference to the robot for correcting a trajectory, such as through language (Bucker et al., 2022; 2023; Sharma et al., 2022), physical interaction (Bajcsy et al., 2017; 2018; Bobu et al., 2021), rankings (Jain et al., 2015) and joystick inputs (Spencer et al., 2020). In this work, we use language as it is the most natural and intuitive way of communicating human preference (Tellex et al., 2020).

Language correction works can be broadly classified into two categories–generating new trajectories (Broad et al., 2017; Sharma et al., 2022; Liang et al., 2023; Zha et al., 2023) and modifying existing ones (Bucker et al., 2022; 2023). The first category, trajectory generation, involves creating new motion plans to enable robots to correct errors to complete tasks. Consider a task where a robot provides feeding assistance to a user. An online correction such as “move 5 cm to the left” when acquiring the food might be provided so the robot can align more accurately with the food morsel before acquiring it. This directs the robot to generate a new trajectory to move to the left for more precise alignment. On the other hand, our work focuses on the second category of corrections, which involves modifying an existing trajectory. This includes relative corrections such as “scoop a larger amount of food”. Such corrections require adjustments to an existing trajectory, requiring an understanding of the initial plan or trajectory. Modifying existing trajectories is important, as human preferences are commonly expressed in relative terms.

Both categories of language corrections face a common challenge: translating natural language to robot actions, also known as language grounding. Language grounding can be categorized into three approaches–semantic parsing to probabilistic graphs, end-to-end learning using embeddings and prompting LLMs to generate code.

### 2.1 Semantic parsing to probabilistic graphs

Earlier works in language correction use a grammatical structure to represent language and ground language by learning the weights of functions of factors (Broad et al., 2017). A probabilistic graphical model (distributed correspondence graph, DCG) is used to ground language to a set of features that relate to the environment and context. Each feature represents a cost or constraint, which a motion planner then optimizes. Given the variety of possible phrases a human can provide to correct the same feature, grounding language using a fixed grammatical structure limits the generalization of this approach. We differ from this approach in how we ground language. Instead of parsing language corrections to phrases with a fixed grammar, we leverage the textual embeddings in LLMs (Devlin et al., 2018; Wang et al., 2020; Brown et al., 2020; Chowdhery et al., 2022) to ground them to textual descriptions of features.

### 2.2 End-to-end learning using embeddings

Prior works have explored LLMs, combining BERT (Devlin et al., 2018) (textual) embeddings and CLIP (Radford et al., 2021) (textual and visual) embeddings to align visual and language representations. Sharma et. al. (Sharma et al., 2022) learn a 2D cost map from CLIP and BERT embeddings, which converts language corrections to a cost map that is optimized using a motion planner to obtain the corrected trajectory. Bucker et. al. goes a step further by directly learning a corrected trajectory from CLIP and BERT embeddings in 2D (Bucker et al., 2022) and 3D (Bucker et al., 2023). Additionally, a transformer encoder obtains geometric embeddings of the object poses and an initial trajectory. A transformer decoder combines textual, visual and geometric embeddings to generate the corrected trajectory. Geometric embeddings have also been combined with textual embeddings to learn a robot policy conditioned on language (Cui et al., 2023), but a shared autonomy paradigm was used to reduce the complexity. These approaches deploy an end-to-end learning approach requiring large multi-modal datasets, including image, text, and trajectory data, which are difficult to obtain. Compared to these works, our proposed approach leverages LLMs to summarize natural language corrections into concise and informative textual representations, thus removing the need for multi-modal training data to ground language corrections.

### 2.3 Prompting LLMs to generate code

Concurrent with our work, advances in Large Language Models (LLMs) have led to recent works (Liang et al., 2023; Huang et al., 2023; Yu et al., 2023; Zha et al., 2023) employing LLMs to generate executable code from natural language instructions. These works mainly focus on generating robot plans or trajectories given a phrase, either by calling pre-defined motion primitives (Liang et al., 2023; Zha et al., 2023) or by designing specific reward functions (Huang et al., 2023; Yu et al., 2023). This approach has shown versatility in handling diverse instructions and constructing sequential policy logic. However, the adoption of these methods is hindered by significant computational costs due to the need for larger, general-purpose models like GPT-4, which cannot be run on standard commercial hardware. Remote execution via API calls is a viable option, but this is limited by the need for a stable internet connection and the rate limits imposed on the APIs, which can hinder real-time applications. Moreover, similar to our approach that integrates hand-crafted templates, the effective use of LLMs for specific task-oriented code generation also requires many in-context examples. In contrast, our approach provides a more cost-effective solution for language understanding in robotic systems by using semantic textual similarity, eliminating the need for extensive prompting or high computational demands. Although our method requires additional modules to process complex language utterances, such as referring expressions and compound sentences, it is a more viable alternative in settings constrained by limited computational resources and internet connectivity. This makes our framework well-suited for practical applications where efficiency is a key consideration.

## 3 Approach

### 3.1 Problem definition

Our goal is to develop an interface that allows users to modify the trajectory of robot manipulators based on their preferences conveyed through natural language corrections. More specifically, the problem can be described as finding the most likely trajectory $\xi^{*}$ given the environment E , the language correction l provided by the human and an initial trajectory $\xi_{0}$ , where $P\left(\xi |E,l,\xi_{0}\right)$ is the probability of a corrected trajectory given the environment, language correction and initial trajectory (Equation 1).

$$\xi^{*}=\underset{\xi }{argmax}P\left(\xi |E,l,\xi_{0}\right)$$ (1)

The environment consists of a set of objects, with each object having two attributes–object name $o_{name}$ and object position $o_{pos}$ , i.e., $E=\left\{\left(o_{name}^{i},o_{pos}^{i}\right)\right\}$ , where i is the object index. The object names and poses in the environment can be obtained using perception algorithms (e.g., Mask R-CNN (He et al., 2017), YOLO (Redmon and Farhadi, 2018) or foundational models (e.g., OWL-ViT (Minderer et al., 2022), Grounding DINO (Liu et al., 2023). In our work, we used Mask R-CNN to get the environment E .

### 3.2 Features

The space of possible trajectories ξ is infinite. However, realistically, it can be bounded by the environment and motion planner constraints (Broad et al., 2017). Therefore, we assume that the language correction l can be mapped to a finite set of features Φ that can be scene-specific or scene-independent. Each feature ϕ∈Φ corresponds to a deformed trajectory, i.e., $\xi =\delta \left(\phi ,\xi_{0},E\right)$ where δ is the trajectory deformation function which we describe in Section 3.4. Thus, we can obtain the most likely trajectory $\xi^{*}=\delta \left(\phi^{*},\xi_{0},E\right)$ from the most likely feature $\phi^{*}$ ^1^. This reduces the problem of finding the most likely trajectory $\xi^{*}$ to the problem of finding the most likely feature $\phi^{*}$ . Thus, our problem can be rewritten as:

$$\begin{array}{c} \phi^{*}=\underset{\phi \in \Phi }{argmax}P\left(\phi |l\right) \end{array}$$ (2)

Where P(ϕ|l) is the probability of feature ϕ given language utterance l . The features can be categorized into two types–scene-specific and scene-independent features. Scene-specific features depend on the objects in the scene, while scene-independent features are not.

As proof of concept, we define two scene-specific and six scene-independent feature templates (Table 1). Scene-specific features are generated online for each object detected in the scene, allowing the approach to generalize to different objects. They are defined based on *object distance*, which is to either increase (obj_distance_increase) or decrease (obj_distance_decrease) the distance to an object. Scene-independent features are based on the three axes in *Cartesian* space, i.e., to move the gripper up (z_cart_increase), down (z_cart_decrease), left (y_cart_decrease), right (y_cart_increase), forward (x_cart_increase) and backward (x_cart_decrease). The space of feature templates is expandable and can be made specific to the robot’s task, as demonstrated in Section 4.5.

**TABLE 1:** Feature Templates (FT) and their Textual Description Templates (TDT).

FT	obj_distance_decrease	obj_distance_increase
TDT	Move closer to {obj}	Move further away from {obj}
	**Stay close to {obj}**	**Stay away from {obj}**
	**Decrease distance to {obj}**	**Increase distance to {obj}**
	Keep a smaller distance from {obj}	Keep a bigger distance from {obj}
		Avoid {obj}

FT	z_cart_decrease	z_cart_increase
TDT	Move closer to table	Move further away from table
	**Stay closer to table**	**Stay away from table**
	Move lower	Move higher
	Move down	Move up
	**Stay down**	**Stay up**
	Go to the bottom	Go to the top
	Down	Stay on the upper part
	Low	

FT	y_cart_decrease	y_cart_increase
TDT	**Stay on the left**	**Stay on the right**
	**Go to the left**	**Go to the right**
	Move left	Move right
	Move more towards the left	Move more towards the right
	Left	Right

FT	x_cart_decrease	x_cart_increase
TDT	**Stay at the back**	**Stay at the front**
	**Go to the back**	**Go to the front**
	Move back	Move front
	Stay back	Stay front
	Move backward	Move forward
	Go behind	

Note: Only the bolded phrases were used for the analysis in Section 4.3.3.

### 3.3 Textual descriptions and optimal feature selection

To compute P(ϕ|l) , we generate a textual description $T_{\phi }$ for each feature ϕ . Each textual description consists of a set of language phrases, which includes commonly used phrases to modify the trajectory for a particular feature. Table 1 shows the textual description templates (TDT) for each feature template (FT). During feature generation for a scene, the {obj} placeholder in scene-specific feature templates will be replaced by the object name $o_{name}$ obtained using object detection, as shown in Figure 2.

We initially defined a few phrases in the textual description templates. If feature matching fails due to out-of-distribution user utterances, additional phrases can be added to the textual description templates easily to improve feature matching. We examine how the phrases in the textual description templates can affect feature matching and performance in Section 4.3.3.

Since there is a one-to-one mapping between the feature and its textual description, Equation 3 can be rewritten as:

$$\begin{array}{c} \phi^{*}=\underset{\phi \in \Phi }{argmax}P\left(T_{\phi }|l\right) \end{array}$$ (3)

To compute $P\left(T_{\phi }|l\right)$ , we leverage large language models (LLMs) to map diverse language phrases to fixed-length vectors called embeddings. To capture the semantic meaning of sentences, we used Sentence Transformers (Reimers and Gurevych, 2019), which is fine-tuned for semantic similarity tasks. We chose the pre-trained all-MiniLM-L6-v2 model (Wang et al., 2020) provided by Sentence Transformers^2^ as it provided us with the best trade-off between speed and performance. Semantically closer language phrases are more likely to have higher cosine similarity between their embeddings. Thus, $P\left(T_{\phi }|l\right)$ can be defined as Equation 4:

$$P\left(T_{\phi }|l\right)\propto \underset{t_{\phi }\in T_{\phi }}{max}q\left(t_{\phi }\right).q\left(l\right)/\|q\left(t_{\phi }\right)\|.\|q\left(l\right)\|$$ (4)

Where *q(x)* is the embedding for a language phrase *x*. Once the most likely feature $\phi^{*}$ is obtained, the trajectory $\xi^{*}$ can be obtained using a deformation function.

### 3.4 Deformation function

A deformation function $\delta \left(\phi ,\xi_{0},E\right)$ modifies a trajectory based on a feature ϕ . First, we calculate the force F to be exerted on each waypoint of the trajectory.

For scene-specific features, i.e., *object distance* features, the force exerted is dependent on the object position $o_{pos}$ and a radius of deformation r . In our experiments, we set r=0.3 , which was determined empirically. We note that r can be set adaptively based on environmental constraints and human preferences, but this will be part of our future work. For waypoints within the radius of deformation r from the object position $o_{pos}$ , a force is applied on the waypoints in the direction of the distance vector between the waypoint and the object. The force is 0 for other waypoints.

For scene-independent features, a force is exerted on all waypoints of the trajectory, where the direction of the force is dependent on the feature.

The trajectory is deformed based on the force calculated on each waypoint: $\delta =\xi_{0}+wF$ , where the weight w changes the magnitude of the deformation. We empirically determined the value of w to be a constant of 1.0 in our experiments, but this can be modified based on the intensity of the language correction in the future.

Finally, the deformed trajectory is passed to a trajectory optimizer to ensure the robot’s kinematic constraints are satisfied.

## 4 Experiments

We conducted simulation and real-world experiments to validate our proposed approach. We hypothesize that:

**H1** Our approach will be able to generalize to natural language phrases and environments with different objects;

**H2** Our approach will deform trajectories at least as accurately as end-to-end methods for trajectory deformation;

**H3** Our approach will obtain higher rankings from users compared to end-to-end methods;

**H4** Our approach will be more interpretable than end-to-end methods.

We compared our approach against LaTTe (Bucker et al., 2023), an end-to-end approach for language corrections that can deform trajectories in 3D space. Experiments were conducted to evaluate the generalization ability of the proposed approach. We also conducted user studies to evaluate the end-users’ satisfaction with the deformed trajectories and to obtain more diverse language utterances. The Nanyang Technological University Institutional Review Board approved the user studies. In the next section, we briefly describe our baseline method, LaTTe.

### 4.1 Baseline

LaTTe first created a dataset consisting of tuples of initial trajectory, language correction, object images, names and poses in the scene, and deformed trajectory. This dataset is then used to train a neural network which can map the initial trajectory, language correction and objects in the scene to a deformed trajectory. For generalization to various objects in the scene and different natural language phrases, pre-trained BERT and CLIP are used to generate embeddings for the language correction and the object images, which are provided as input to the neural network. We used the model provided by the authors^3^, trained on 70k samples for our experiments. Before generating the deformed trajectory using the trained model, a locality factor hyper-parameter must be set, determining the range of desired change over the trajectory. In our user studies, we set the locality factor hyper-parameter to be 0.3, the mean value in their dataset.

### 4.2 Generalization experiments

To evaluate the ability of our approach to generalize to different object configurations, trajectories and corrections, we used the dataset that was originally employed to train the LaTTe. The LaTTe dataset includes 100k samples, with object names sampled from the Imagenet dataset. The full dataset contains three types of trajectory modifications–*Cartesian* changes, *object distance* changes and *speed* changes. We removed the samples related to speed changes in our evaluation as we did not include speed features in our templates, resulting in 65,261 samples. We set the locality hyper-parameter for LaTTe by referencing the value provided in each sample in the dataset.

#### 4.2.1 Evaluation metrics

To evaluate the accuracy of the deformed trajectories, LaTTe compared the similarity between the deformed trajectory using their method and the ground-truth output trajectory in the dataset using metrics like dynamic time warping (DTW) distance. However, this may not accurately capture the correctness of the trajectory modification. For example, even if the trajectory change occurs in a direction contrary to the intended one, the DTW distance may still register as small. Additionally, relying on a single ground-truth trajectory for comparison is problematic, as multiple valid trajectories can achieve the correct deformation.

**FIGURE 3:**
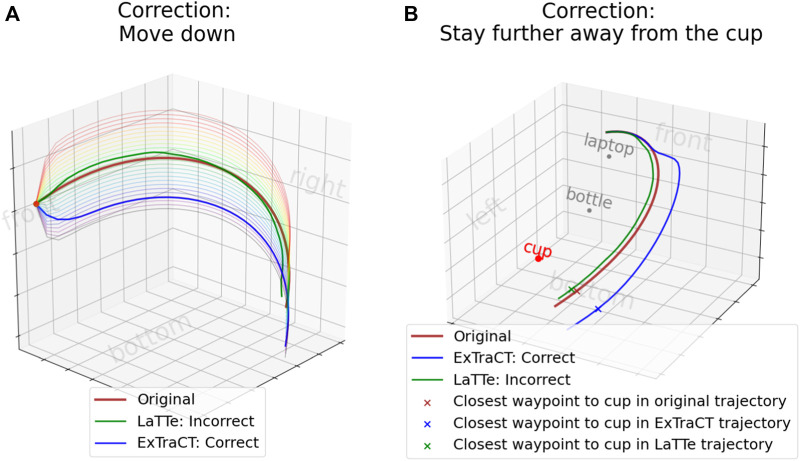
Accuracy evaluation. The green trajectory shows an incorrect deformation, while the blue trajectory shows a correct deformation. **(A)** Cartesian changes - we sampled trajectory deformations with varying weights, as shown by the different colored trajectories, which affect the intensity of deformation. **(B)** Object distance changes–we obtained the waypoints in the original and deformed trajectories and compared the change in distance relative to the target object.

Thus, we developed a way to assess the performance of trajectory deformations based on language. Our approach evaluates the accuracy of deformed trajectories by considering the type of correction applied, as shown in Figure 3.

For corrections involving *Cartesian* changes, we first sampled a range of trajectory deformations of different intensities, i.e., by varying the value of w in the deformation function (Section 3.4) from −2.5 to 2.5. Dynamic time warping (DTW) distance was then used to find the weight that best represents the deformed trajectory. A deformation is deemed correct if the weight aligns with the intended change direction. For example, a positive weight should correspond to a positive change (i.e., increase), and a negative weight should correspond to a negative change (i.e., decrease).

For corrections involving *object distance* changes, we measure the accuracy by comparing the distance between the object and the waypoint closest to the object in both the original and deformed trajectories. The key metric is whether the modified trajectory brings the waypoint closer to or further from the target object, which can be quantified without sampling multiple trajectories. A deformation is deemed correct if, for a positive change, the waypoint in the deformed trajectory is further from the object, and for a negative change, it is closer to the object.

To facilitate a direct comparison with the findings reported in LaTTe, we also evaluated the performance in terms of the similarity between the deformed trajectory of each approach and the ground-truth output trajectory in the dataset using the dynamic time-warping (DTW) distance. While LaTTe reported both the DTW distance and the discrete Frechet distance (DFD), we opted for the DTW distance as it provides a more comprehensive similarity measure, accounting for variations in the overall shape of the trajectory and the timing of specific waypoints.

#### 4.2.2 Results

Table 2 presents the evaluation results, where our approach outperformed the baseline, showing support for **H1** and **H2**. To determine whether the type of change affects the performance, we analysed the results based on the change category, i.e., *object distance* changes and *Cartesian* changes. Even though both approaches have comparable performance for *Cartesian* changes, we see that the performance of LaTTe degrades for *object distance* changes, with an accuracy of only 54.5% , which is approximately equivalent to random chance.

**TABLE 2:** Generalization experiment results.

Approach	Overall		Object Distance Changes		Cartesian Changes	
	Accuracy	DTW	Accuracy	DTW	Accuracy	DTW
ExTraCT	**89.23%**	**2.6386**	**86.10%**	**3.0600**	92.27%	**2.2100**
LaTTe	73.37%	3.4099	53.48%	3.7499	**92.64%**	3.0804

Bolded values indicate the best performance.

#### 4.2.3 Analysis of failure cases in LaTTe

**FIGURE 4:**
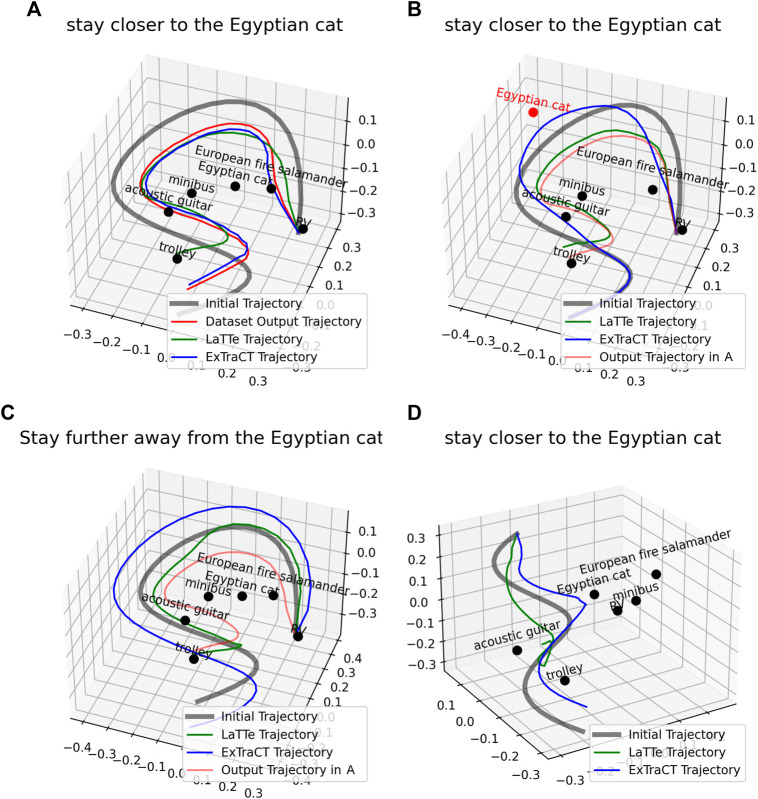
Changes in the deformed trajectory using **(A)** a sample in LaTTe’s dataset **(B)** a change in the target object pose **(C)** a change in the language correction that conveys an opposite meaning **(D)** a change in the initial trajectory. The deformed trajectory by LaTTe is inaccurate for **(B), (C)** and **(D)**, while ExTraCT produces a correct trajectory deformation for all cases.

To better understand the degraded performance of LaTTe for *object distance* changes, we performed qualitative analysis on LaTTe by picking a random sample in LaTTe’s dataset (Figure 4A) and modifying the target object pose (highlighted in red, Figure 4B), language correction (Figure 4C) and initial trajectory (Figure 4D). The trajectories deformed by LaTTe shown in Figure 4 demonstrate that these changes did not result in an expected change in the deformed trajectory. Figures 4B, C show a similar (but incorrect) deformed trajectory to the original sample, while Figure 4D shows the deformed trajectory in the opposite direction to the correction. On the other hand, ExTraCT deforms the trajectories correctly in all these cases.

To understand why a change in the language correction (Figure 4C) did not result in a correct change in the deformed trajectory, we analysed the embeddings of the language correction. We used Sentence Transformers (Reimers and Gurevych, 2019) to find the top eight sentences with the closest semantic similarity in LaTTe’s dataset. Sentences that convey opposite meanings but have high lexical similarity, such as “stay closer to the Egyptian cat” had a high cosine similarity score to “stay further away from the Egyptian cat”, as highlighted in Table 3. This makes learning trajectory deformations from textual embeddings difficult, as the BERT embeddings used may not always capture the semantic meaning of the corrections.

**TABLE 3:** Top 8 Sentences with Highest Similarity Scores for ”Stay further away from the Egyptian cat” in LaTTe’s Dataset.

Sentence	Similarity Score
Stay further away from the Egyptian cat	1.00
Stay a lot further away from the Egyptian cat	0.98
**Stay closer to the Egyptian cat**	**0.91**
Walk a lot further away from the Egyptian cat	0.90
Walk a bit further away from the Egyptian cat	0.89
**Stay a lot closer to the Egyptian cat**	**0.88**
**Stay very closer to the Egyptian cat**	**0.88**
**Stay a bit closer to the Egyptian cat**	**0.87**

Note: Bolded phrases indicate sentences with opposite meanings.

Unfortunately, for the cases where we modified the target object pose (Figure 4B) and the input trajectory (Figure 4D), it was difficult to understand why failures occurred. Failures could arise from various factors, such as errors in embedding geometrical information like trajectories and object configurations and insufficient training data. The lack of transparency makes identifying and rectifying specific issues difficult, motivating our separation of the problem into two distinct phases–language understanding and trajectory deformation.

#### 4.2.4 Analysis of failure cases in ExTraCT

All the failure cases in ExTraCT can be attributed to incorrect feature mapping. For example, “Go to the upper part” was incorrectly mapped to z_Cartesian_decrease as the most similar phrase was “Go to the bottom”. “Drive a lot closer to the meat market” was incorrectly mapped to meat market_distance_increase as the closest phrase was “Stay a lot further away from meat market”. These errors occurred as the embeddings from large language models (LLMs) may not always capture the nuanced semantic meanings of language phrases (Table 3).

To improve the language understanding capabilities of our system, we can either fine-tune the existing language model for our application or employ a larger LLM with better semantic understanding. Another way to improve performance is to include previously mismatched phrases in our textual description templates. In Section 4.3.3, we show how expanding the textual description templates can improve performance. Note that for this evaluation, we deliberately did not include all the phrases in LaTTe’s dataset in our template descriptions, as that would naturally lead to an exact match in the sentence, which would not realistically reflect the true language understanding capabilities of our approach.

### 4.3 User studies in simulation

**FIGURE 5:**
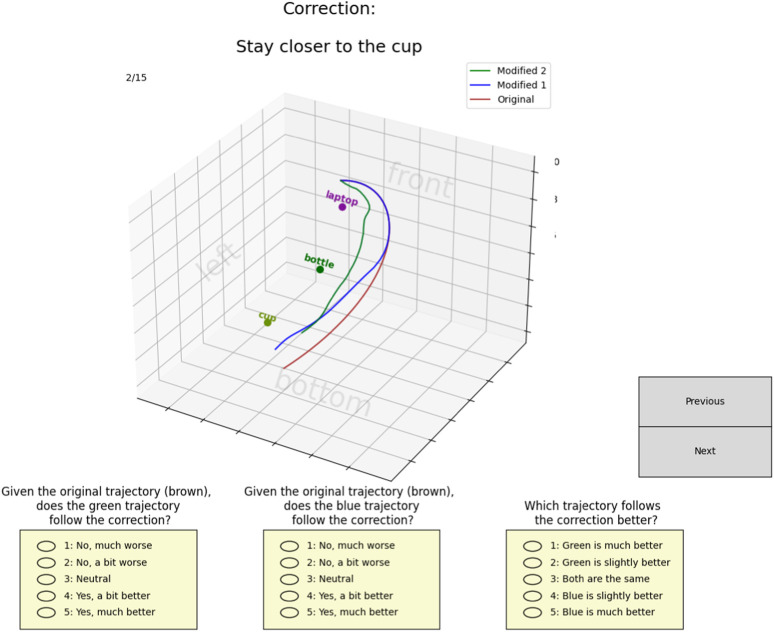
Interface for the simulation study showing scene 1. The modified trajectories are displayed on the interface simultaneously for better comparison. The green modified trajectory is by LaTTe, while the blue modified trajectory is by our approach.

We recruited 15 subjects (8 male, seven female) for the simulation study. Out of these participants, six individuals did not have a background in robotics. The study interface (Figure 5) (modified from LaTTe), displayed the initial trajectory, the modified trajectory using LaTTe and the modified trajectory using our method (ExTraCT) at the same time. The trajectories were displayed to the users in 3D and subjects could interact with the plots to change the view. The participants were not informed which method was used to deform the trajectories, and the modified trajectories were labelled only with ”1″ and ”2”. The labels were kept consistent throughout the experiment so that we could obtain the subjects’ overall rankings and preferences for each method across different scenes.

**FIGURE 6:**
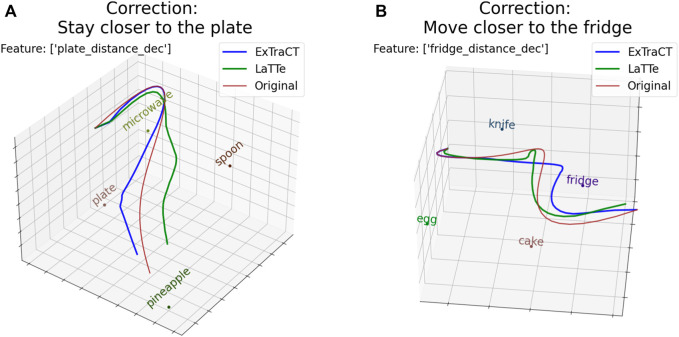
The deformed trajectories and features matched for **(A)** scene two and **(B)** for scene 3. Note that the modified trajectory by LaTTe opposes the corrections provided in these examples.

Three scenes were presented to each subject, as shown in Figures 5, 6. Different household objects were placed at randomized locations for each scene. The objects in scenes one and two were selected from LaTTe’s dataset, while those in scene three were randomly selected and out of LaTTe’s dataset. Since there were no images of the objects, no CLIP image embeddings were used for LaTTe. Instead, CLIP textual embeddings of the object names were used for LaTTe to identify the correct target object.

There were five language corrections for each scene, where three of the corrections were given by us (for consistency across subjects and to familiarize subjects with the types of corrections), and two of the corrections were given by the subjects. Subjects were free to provide any language corrections, after which both approaches would modify the trajectory. After each correction, subjects had to rank their agreement on how well each method modified the trajectory and which trajectory they preferred, if any. Specifically, for each method, they had to choose whether the deformed trajectory was 1) Completely wrong, 2) Somewhat wrong, 3) Neutral, 4) Somewhat correct, or 5) Completely correct. After each trajectory deformation and at the end of the experiments, they had to compare both methods. For this, they had to choose whether 1) Method one was much better, 2) Method one was a bit better, 3) Both the methods were the same, 4) Method two was a bit better, or 5) Method two was much better. At the end of the study, subjects were asked to elaborate on which method they preferred and why. We also measured the performance based on the accuracy of the trajectory deformations, as outlined in Section 4.2.1.

#### 4.3.1 Results

Table 4 shows the results of our user study, showing better accuracy and preference for our method, supporting **H2** and **H3**. A Wilcoxon signed-rank test showed a significant difference in the rankings between our method and LaTTe (p<0.0001) on how well the trajectory followed the correction. When comparing the two methods after each correction, subjects rated that our method was better 85.5% of the cases, LaTTe was better 6.2% of the cases, and there was no difference for 8.0% . When asked at the end of the experiments, all the subjects preferred our method, with 33.3% of subjects rating that our method was “slightly better”, and 66.7% of subjects rating that our method was “much better” than LaTTe.

**TABLE 4:** Simulation user study results.

Approach	Overall		Object Distance Changes		Cartesian Changes	
	Accuracy	Mean User Rank	Accuracy	Mean User Rank	Accuracy	Mean User Rank
ExTraCT	**88.00%**	**4.471** ± 0.062	**96.53%**	**4.521** ± 0.0668	**100.00%**	**4.776** ± 0.060
LaTTe	55.56%	2.840±0.0790	47.22%	2.722±0.0988	94.83%	3.207±0.136

Bolded values indicate the best performance.

The higher preference for our method could be largely attributed to the accuracy of our approach, with many subjects stating that ExTraCT “follows [the] correction more”. LaTTe did not deform trajectories according to the corrections for many cases, such as in Figures 6A, B, where the deformed trajectories were opposite to the corrections.

#### 4.3.2 Failure cases

There were 12.00% of failure cases with ExTraCT. Due to the explainability of our approach, we can analyse the feature matching and confidence score for each failure case (**H4**). We labelled the correct feature(s) for corrections within our feature space. For example, “Move below the fridge”, which contains a directional component for an object, is out of our feature space. These failures can be attributed to a lack of a correct deformation function. There were 12 such corrections (5.33%) , which we removed from subsequent analysis.

The remaining 15 failures could be attributed to two reasons. Of these, 10 failures were due to multiple trajectory modification features within a single correction, such as “Move away from bottle and then closer to cup”. Since we assumed that each correction only contains one feature, such failures were expected. Five failures were due to incorrect feature mapping, i.e., “Stay further away from the pineapple” was incorrectly mapped to pineapple_distance_decrease, as the most similar phrase was “Stay close to pineapple”; and “Move to the cake” was mapped to cake_distance_increase since the most similar phrase was “Move further away from cake”. These failures highlight the limitations of using Sentence Transformers for capturing semantic nuances in language corrections. Fine-tuning the model with in-domain data could improve the performance.

#### 4.3.3 Effect of textual description templates on performance

We investigated the impact of the size of textual descriptions (number of phrases in each textual description) and phrase selection on performance. We selected phrases less frequently provided in the simulation experiments from the original set of textual description templates. The resulting textual description templates contained only phrases, highlighted in bold in Table 1. Using this smaller set of phrases, we examined the features matched for the 59 unique language corrections from the simulation study. With a smaller set of language phrases, the number of errors in terms of feature mapping remained at 6, but there were three instances of low confidence. Even though the number of errors was the same, the phrases with incorrect feature mapping differed.

We also demonstrate how we can easily add phrases to our textual description templates and improve the system’s performance. We added the following phrases that were incorrectly mapped to the feature during the simulation study – “Stay further away from the {obj}” to obj_distance_inc.; “Move towards {obj}”, “Move to {obj}” to obj_distance_dec; and “Move to the right” to y_cart_inc. After adding the phrases with incorrect feature mappings to the textual description templates, there were no more inaccurate feature mappings.

While pre-trained LLMs demonstrate promise in semantic similarity matching, their ability to generalize to the full diversity of language is influenced by the comprehensiveness and size of their training data. Using a larger pre-trained LLM could improve performance, but its computational demands can also increase significantly. Our approach’s explainability provides valuable insights to enhance the system’s performance. We demonstrate that expanding the phrases in the textual description templates can improve language grounding to features, improving our approach’s generalizability to language variations.

### 4.4 User studies with real arm

We also conducted user studies using the xArm-6 robotic arm (UFactory, China) with the xArm two-finger gripper. An Intel Realsense Depth Camera D435 was mounted on the xArm-6 gripper to capture images of the scene to estimate the location of the objects. Additionally, for LaTTe, the bounding boxes of the objects were also estimated to obtain the CLIP embeddings of the objects. Object detection was performed using Mask R-CNN (He et al., 2017) in our experiments. TrajOpt (Schulman et al., 2014) was used to optimize the trajectory based on the robot kinematic constraints.

We recruited five subjects (2 male, 3 female) for a within-subject study, where each subject evaluated two methods–our approach (ExTraCT) and LaTTe.

#### 4.4.1 Setup and protocol

**FIGURE 7:**
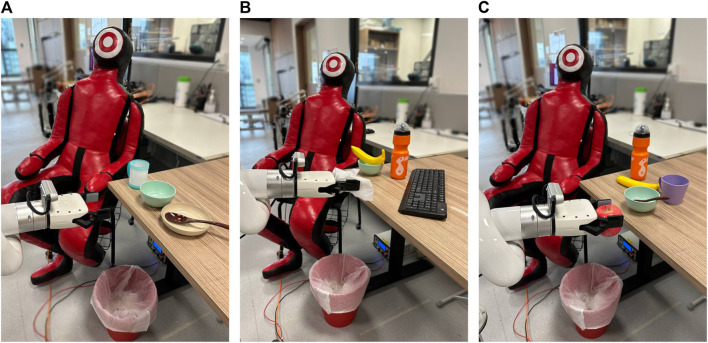
Setup of the experiments using the xArm-6 manipulator for **(A)** scene 1 **(B)** scene two and **(C)** scene 3. For safety, users were seated outside the robot’s workspace and a dummy was placed at the scene.

There were three scenes with different objects on the tables. Figure 7 shows the scenes used for real arm experiments. The object types, number of objects and object positions varied across the scenes. For the first scene, we provided the language corrections for consistency across subjects and also to familiarize the subjects with the types of corrections. We did not explicitly set a task for the robot to complete in Scene 1. The subjects had to rate the performance of the following corrections for both methods – ”Go down”, ”Keep a bigger distance away from bowl”, ”Stay closer to spoon”.

For the next two scenes, subjects had to use language corrections to modify a trajectory to complete tasks. Subjects were informed that they could only give one correction at a time, with three corrections required. The second scene required subjects to throw trash into the bin while avoiding the food items and objects on the table. The third scene required subjects to bring an apple to a dummy for handover while staying away from the bowl and staying closer to the table. In both scenes, subjects also had to modify the trajectory’s final waypoint to move closer to the bin or the dummy. The language corrections were manually typed into the computer running both methods. Subjects completed both methods for each scene before moving to the next scene.

The order of the methods was randomized across subjects to counteract the effects of novelty and practice. Subjects filled out a qualitative survey after each task and each method, rating on a scale of one–five whether the modified trajectory followed the language correction. At the end of the study, subjects were asked to elaborate on their preferred method and why. We also measured the accuracy of the trajectory deformations using the same method as in Section 4.2.1.

#### 4.4.2 Results

The real-arm study showed similar results to the simulation study (Table 5), where ExTraCT deformed trajectories more accurately than LaTTe (**H2**). A Wilcoxon signed-rank test was performed on the user rankings. Statistically significant differences were found, with our trajectory deformations following the corrections better (p<0.0001) , showing support for **H3**. Subjects generally preferred our method, with 80% of subjects rating our method slightly better or much better than LaTTe. Subjects preferred our method because it “captures the user’s intention better” and has a “greater accuracy”.

**TABLE 5:** Real arm experiment results.

Method	Accuracy	Mean User Rank
ExTraCT	97.78%	4.38±0.12
LaTTe	66.67%	3.16±0.20

### 4.5 Application in assistive feeding

**FIGURE 8:**
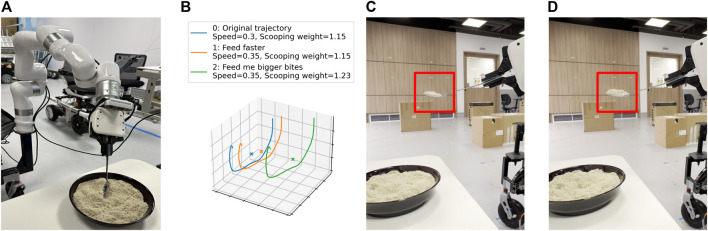
**(A)** Setup for assistive feeding using a spoon. **(B)** The change in scooping trajectories after a language correction is provided. **(C)** Original bite size. **(D)** Bite size after the correction “Feed me bigger bites” was provided.

We deployed our framework in an assistive feeding task to show that our framework is versatile and can be applied across diverse scenarios. To tailor our framework for this context, we defined two features–*bite size* and *feeding speed*. The features and textual descriptions are shown in Table 6. We show some sample interactions on how the amount of food scooped can be modified using language in Figure 8. The modularity of our framework allows different trajectory modification methods to be used depending on the task’s specific requirements. In this case, we used parameterized dynamic motion primitives (DMP) to modify the bite size.

**TABLE 6:** Features (F) and their Textual Descriptions (TD) for Assistive Feeding.

F	bitesize_increase	bitesize_decrease
TD	I want a bigger bite	I want a smaller bite
	Increase the spoonful size	Decrease the spoonful size
	I want a larger bite next	I want a smaller bite next
	I want more food	I want less food
	Increase bite size	Decrease bite size

F	speed_increase	speed_decrease
TD	Move faster	Move slower
	Increase speed	Decrease speed
	Too slow	Too fast

Figure 8B displays the variations in scooping trajectories based on language corrections. After the correction “Feed faster” was provided, the scooping speed increased. Similarly, following the correction “Feed me bigger bites”, the scooping trajectory was modified by scaling up the weight of the DMP, resulting in a larger bite size, as seen in Figure 8D.

## 5 Conclusion and discussion

In conclusion, we propose ExTraCT, a modular trajectory correction framework. ExTraCT creates more accurate trajectory modification features for natural language corrections, which is important for safety and building trust in human-robot interaction. Our proposed architecture uses pre-trained LLMs for grounding user corrections and semantically maps them to the textual description of the features.

Our approach combines the strengths of hand-crafted features in trajectory deformations to generalize to different object configurations and initial trajectories and the language modelling capabilities of LLMs to handle language variations. By separating the problem of language understanding and trajectory modification, we have shown improvements in interpreting and executing language corrections, even for non-templated language phrases. The transparency of our approach allowed us to understand the root causes of failures, whether in language understanding or the trajectory deformation process, enabling more targeted improvements to the system.

**FIGURE 9:**
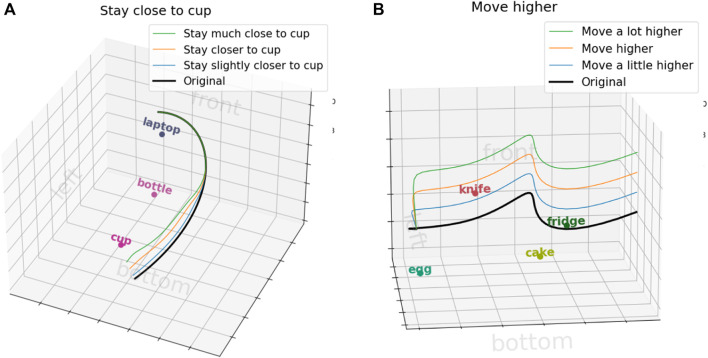
Trajectory modifications based on different intensities in the language utterance. **(A)** Different intensities for object distance changes. **(B)** Different intensities for Cartesian changes.

This work is just a step towards understanding how explicitly obtaining the features for trajectory modification can help provide a more explainable and generalizable approach. In this work, our focus was on comparing our approach against an end-to-end method for language corrections with only a single feature. As such, our feature space is limited, and we cannot extract multiple features from a single correction. While our current implementation allows a chain of corrections, this approach may be inefficient for handling the natural complexity of language. Future work aims to increase our feature space and handle more complex language utterances, such as different intensities of trajectory modifications, compound sentences and referring expressions. Preliminary work has been done to identify the degree of change in language corrections by utilizing part-of-speech (POS) tagging and dependency tagging. These techniques effectively identify the modifiers in the language utterances, allowing us to modify the degree of change in the trajectory, as demonstrated in Figure 9. However, the intensity of modification will also be influenced by the scene context and initial trajectory. For example, consider the instruction, ”Move slightly closer to the cup”. The desired change in distance depends on the initial distance to the cup. If the cup is 10 cm away, ”slightly closer” might imply a 2 cm adjustment, while a 30 cm distance might imply a 10 cm adjustment. To handle compound sentences, we believe decomposing them into simpler units offers a promising approach for a more generalizable solution. This decomposition can be achieved using standard natural language processing (NLP) methods or by leveraging frameworks like Frame Semantics (Fillmore et al., 2006; Vanzo et al., 2020) to identify multiple frames.

Another direction is to look into bi-directional communication between the robot and the user, allowing the robot to ask the user for clarifications in cases of uncertainty (i.e., low confidence in feature mapping), as has been demonstrated in Shridhar et al. (2020); Yow et al. (2024). This is important as we expand our feature space, as more features and textual descriptions could lead to cases where the same utterance could map to multiple features. The ability to integrate uncertainty handling in our framework also offers an additional advantage over end-to-end methods.

We hope our framework can provide a more transparent approach to learning human preferences, which can serve as a basis for transferring human preferences across different contexts.

## Data Availability

The original contributions presented in the study are included in the article/supplementary material, further inquiries can be directed to the corresponding author.

## Footnotes

1 We assume that a given language correction will correspond to only one feature. Practically, a complex language correction can map to multiple features, but the focus of this work is on comparing our proposed approach against an end-to-end method, which could only handle one feature at a time. We will address this issue in the future.

2
https://www.sbert.net/docs/pretrained_models.html

3
https://github.com/arthurfenderbucker/LaTTe-Language-Trajectory-TransformEr
